# Are There Differences in Skin Autofluorescence-Measured Advanced Glycation End-Product Levels between Chronic Kidney Disease and Kidney Transplant Recipients?

**DOI:** 10.3390/diagnostics14131383

**Published:** 2024-06-28

**Authors:** Josipa Radić, Marijana Vučković, Hana Đogaš, Andrea Gelemanović, Andrej Belančić, Mislav Radić

**Affiliations:** 1 Department of Internal Medicine, Division of Nephrology and Dialysis, University Hospital of Split, 21000 Split, Croatia; josiparadic1973@gmail.com (J.R.); mavuckovic@kbsplit.hr (M.V.); hana.dogas@gmail.com (H.Đ.); 2 Department of Internal Medicine, School of Medicine, University of Split, 21000 Split, Croatia; 3 Mediterranean Institute for Life Sciences (MedILS), 21000 Split, Croatia; andrea.gelemanovic@gmail.com; 4 Department of Clinical Pharmacology, Clinical Hospital Centre Rijeka, Krešimirova 42, 51000 Rijeka, Croatia; a.belancic93@gmail.com; 5 Department of Basic and Clinical Pharmacology with Toxicology, Faculty of Medicine, University of Rijeka, Braće Branchetta 20, 51000 Rijeka, Croatia; 6 Department of Internal Medicine, Division of Rheumatology, Allergology and Clinical Immunology, University Hospital of Split, 21000 Split, Croatia

**Keywords:** advanced glycation end products, chronic kidney disease, kidney transplantation, nutritional status

## Abstract

The aim of this cross-sectional study was to evaluate the differences in the levels of advanced glycation end products (AGE) between patients with chronic kidney disease (CKD) and kidney transplant recipients (KTRs) and to investigate the risk factors for the AGE levels in each group of these patients. There were 217 participants total, of which 99 (45.6%) were KTRs and 118 (54.4%) had CKD. Data on the levels of AGE, body mass composition, anthropometric parameters, central and peripheral blood pressure, and clinical and laboratory parameters were gathered for each study participant. The AGE values of the CKD and KTRs groups did not differ from one another. In both groups, a lower estimated glomerular filtration rate, male sex, and older age were positive predictors for increased AGE values. Furthermore, higher levels of AGE were linked to lower central systolic blood pressure (cSBP) in the CKD group, whilst, in the KTRs group, higher levels of AGE were linked to a shorter time since kidney transplantation (KTx), more years of dialysis prior to KTx, lower levels of trunk visceral fat, the presence of arterial hypertension, and the absence of prescriptions for the antihypertensive medications urapidil and angiotensin II receptor blockers. Further studies are needed to better understand the above associations. Consequently, a personalised multidisciplinary approach to assess the cardiovascular as well as dietary and lifestyle risk factors to reduce the AGE levels in both KTRs and CKD patients may be implemented.

## 1. Introduction

Advanced glycation end products (AGE) are long-lived chemical intermediates formed by the reactions of chemically reactive sugars with proteins, lipids, and nucleic acids [1,2]. Reduced kidney clearance and accelerated AGE production [3] are among the most likely reasons for the increased concentrations of AGE in individuals with chronic kidney disease (CKD). In vitro experiments suggest the enhanced formation of AGE, mainly Nepsilon-(Carboxymethyl) lysine (CML) and pentosidine, and their reactive precursors in uremic patients when compared to healthy controls. The latter indicates the presence of circulating factors that promote and stimulate the production of the aforementioned molecules in the blood of individuals with impaired kidney function [4]. Furthermore, the skin collagen fluorescence, used as a biomarker of the AGE burden, was significantly increased in non-diabetic CKD patients [3,5] as well as a notable predictor of CKD progress [6]. An inverse correlation was determined between the AGE levels and the estimated glomerular filtration rate (eGFR) as a kidney function marker [7], whereas positive correlations were found with the CKD incidence and mortality in individuals affected by both CKD and diabetes mellitus [8]. Evidence from recent studies has highlighted the crucial role of AGE in the development of the complications associated with CKD, the most common among them being anaemia [9], atherosclerotic cardiovascular disease [10], left ventricular hypertrophy, and heart failure [11,12].

Furthermore, even though kidney transplantation (KTx) restores the eGFR, the AGE remain disproportionally high compared to the eGFR increase, suggesting that other factors may impact AGE production (e.g., increased carbonyl stress and oxidative stress) [13,14]. KTx by itself is associated with higher levels of low-grade chronic inflammation, which is indirectly related to an increase in AGE [15]. An increase in the circulating AGE is also associated with impaired renal clearance and the increased formation of kidney transplant recipients (KTRs), which activate multiple intracellular signalling pathways, leading to an increased risk of cardiovascular disease (CVD) [16]. Thus, some research suggests that AGE may contribute to the development of CVD and chronic kidney transplant failure [17]. One study found a significant decrease in the AGE levels in KTRs compared to dialysis patients (285 KTRs vs. 32 dialysis patients vs. 231 healthy subjects); however, compared to healthy subjects, the AGE remained significantly higher. In summary, it appears that KTx cannot fully repair dialysis patients’ increased AGE levels [14]. Furthermore, immunosuppressive medications promote an increase in AGE levels, which in turn causes enhanced oxidative stress in KTx [15].

Another cohort study found that the AGE levels were similar in KTRs and stage 3 CKD patients. In a cross-sectional study of KTRs and CKD patients, the authors demonstrated that the accumulation of AGE (as measured by skin autofluorescence) is less in KTRs as compared with ESRD patients but comparable to the levels in patients with CKD stage 3 [18]. Furthermore, a recent review on the AGE accumulation in CKD states that, independent of hyperglycaemia, the tissue accumulation of AGE contributes to the progression of diabetic nephropathy. Diabetic nephropathy being the number one cause of CKD makes this finding extremely important in further evaluation and studies on AGE in diabetic patients with CKD [19].

Given the complex interplay of factors contributing to the AGE levels in both CKD and KTRs patients and the differences in the clinical aspects of these groups, the aim of the present study was to assess the difference in the AGE levels between CKD patients and KTRs and to investigate the risk factors for the AGE levels in each group of these patients.

## 2. Materials and Methods

### 2.1. Study Design and Population

This cross-sectional study was carried out at the Department of Internal Medicine, Division of Nephrology and Dialysis, University Hospital of Split, Croatia, in the period between July and December 2019. A total of two hundred and seventeen (217) adult participants, of whom 99 (45.6%) received a kidney transplant and 118 (54.4%) had CKD, were recruited during their regular visits to the nephrologist. Regarding the KTRs population, we included participants with stable kidney function and no signs of acute rejection. All participants (both CKD and KTRs groups) were subject to the following exclusion criteria: condition requiring hospitalisation; dialysis-dependent patients; implanted pacemaker or cardioverter-defibrillator; stents or limb amputation; existing acute infection; existing active underlying malignant disease; COVID-19 recovery in the past three months; participants without body composition and AGE measurements; and refusal to participate in the study.

All participants were informed about the purpose of the study, as per good clinical practice standards, and provided written and oral consent.

### 2.2. Advanced Glycation End Products (AGE) Measurement

A non-invasive device (AGE Reader mu, Diagnostics Technologies BV, Groningen, The Netherlands), based on skin autofluorescence, was used to measure AGE. All measurements were performed on the participant’s forearm, more precisely on the skin site without visible abnormalities, which was previously cleaned with medical alcohol and placed on the device. Three consecutive measurements were taken for each study participant, and the average value was calculated [20].

### 2.3. Body Composition and Anthropometric Parameters

MC-780 Multi Frequency Segmental Body Analyzer (Tanita, Tokyo, Japan) was used to assess body composition. The device is based on bioelectrical impedance and provides data for the following parameters: body mass (kg), fat mass (% and kg), fat-free mass (kg), muscle mass (% and kg), visceral fat level, metabolic age, phase angle (°), skeletal muscle mass (% and kg), and trunk fat mass (kg). All participants were advised beforehand to follow the instructions provided by the manufacturer: to empty the bladder before measurement, not to take any food or liquid at least 3 h before measurement, and not to consume alcohol or have vigorous physical activity for at least one day before measurement [21].

Prior to body composition measurement, body height was measured using a stadiometer for each study participant, and body mass index (BMI) was calculated. The flexible and non-stretchable measuring tape was used to assess waist circumference (WC).

### 2.4. Blood Pressure Measurements

For each study participant, Agedio B 900 (IEM, Stolberg, Germany) device, operating on the principle of oscillometry, was used to assess central and peripheral blood pressure as well as arterial stiffness. Obtained data included pulse wave velocity (PWV; m/s), augmentation index (AiX; %), peripheral systolic blood pressure (pSBP), peripheral diastolic blood pressure (pDBP), peripheral mean arterial pressure (pMAP), peripheral pulse pressure (pPP), central systolic blood pressure (cSBP), central diastolic blood pressure (cDBP), central mean arterial pressure (cMAP), central pulse pressure (cPP), and heart rate (HR).

The upper arm circumference was measured to select and position the right-sized cuff. All measurements were performed according to the User Manual [22]. The participants were instructed to empty the bladder prior to measurement, to remain in a sitting position with legs not crossed, to relax as much as possible, and to refrain from speaking during the measurement.

### 2.5. Medical History, Clinical and Laboratory Parameters

Data about time since KTx, presence of diabetes mellitus, arterial hypertension, CVD and cerebrovascular (CVA) disease/events, and medical therapy were obtained through the participants’ medical records examination.

For the study purpose, all participants underwent the usual peripheral blood sampling in fasting conditions. Blood samples were collected by the trained project nurse on the same day as body composition, blood pressure, and AGE measurements. The following data were collected: erythrocyte count (E; ×10^12^/L), haemoglobin (Hb; g/L), mean cellular volume (MCV; fL), serum albumin (g/L), C-reactive protein (CRP; mg/L), urea (mmol/L), creatinine (mmol/L), eGFR (mL/min/1.73 m^2^) using Chronic Kidney Disease Epidemiology Collaboration (CKD-EPI), uric acid (mmol/L), phosphates (P; mmol/L), sodium (Na; mmol/L), potassium (K; mmol/L), calcium (Ca; mmol/L), fasting blood glucose (FBG; mmol/L), total cholesterol (mmol/L), high-density lipoprotein (HDL) cholesterol (mmol/L), low-density lipoprotein (LDL) cholesterol (mmol/L), and triglycerides (mmol/L). The blood samples were collected in the standard test tubes without additives in the Laboratory of Medical Diagnostics and Biochemistry at the University Hospital of Split, Croatia, and were centrifuged on the HERMLE Z400 centrifuge model (Hermle Labortechnik GmbH, Wehingen, Germany). The complete blood count was obtained using a haematology analyzer (Advia 120, Siemens, Erlangen, Germany). The Jaffe method was used for the creatinine measurement, and standard laboratory methods were applied for the determination of urea, uric acid, serum albumin, phosphates, and CRP concentrations.

### 2.6. Statistical Analysis

Normality of the data was assessed with Shapiro–Wilk test, and, if the data were normally distributed, they were presented with mean and standard deviation (SD), while, in the cases of non-parametric distribution, data were presented with median and interquartile range (IQR). Categorical data were presented as absolute and relative frequencies. To test the differences among groups, chi-squared test was used for categorical data, while *t*-test and Mann–Whitney U test for parametric and non-parametric numerical data, respectively. To assess the correlation between AGE and other numerical variables, Spearman’s rank correlation analysis was performed. Finally, to find predictors for AGE, first, a univariate linear regression adjusted for age, sex, and eGFR was performed. Variables with *p*-value lower than 0.2 and with variance inflation factor (VIF) lower than 4 were selected and multivariate stepwise linear regression with both backward and forward selection was performed. Apart from the univariate linear regression, results of all other statistical tests were assigned as significant if *p*-value was lower than 0.05. What is more, all statistical tests were two-tailed and had a 95% CI. Statistical analyses were performed in R version 4.0.0 [23].

## 3. Results

**Figure 1:**
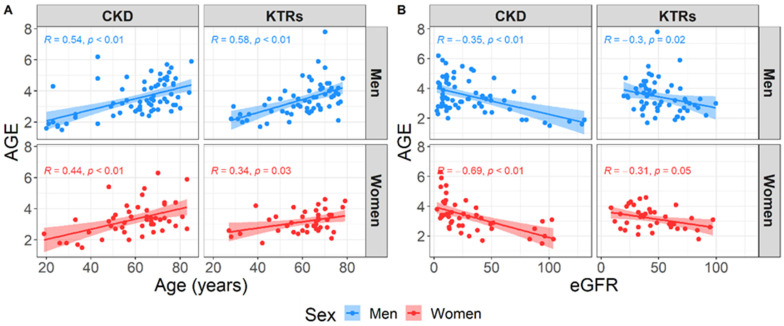
Correlation pattern between the advanced glycation end products (AGE) and age (**A**) or estimated glomerular filtration rate (**B**) stratified by sex.

The total study population consisted of 217 participants, of which 118 suffered from CKD (CKD group; 54.4%), while 99 have undergone KTx (KTRs group; 45.6%). Two groups of patients did not differ in their main demographic characteristics; 40% were women, and the median age of all the participants was 64 years (IQR = 18). The median time that passed from the time point when the KTRs group received a kidney transplant was 7.5 years (IQR = 10). There were some differences in comorbidities between the two groups; arterial hypertension and CVA were significantly more frequent among the KTRs (89.9% vs. 71.8%, *p* = 0.002; 12.1% vs. 2.5%, *p* = 0.012; respectively), while diabetes mellitus was more frequent among the CKD group (33.9% vs. 23.2%, *p* = 0.115) (Table 1), although the difference did not reach a significant level. The two groups of participants differ in most of the measured laboratory parameters (Table 1). The median AGE value was in general quite high, at 3.3 (IQR = 1.3), for all the study participants. When dividing the AGE into categories, which are adjusted for age and gender and correspond to grouping the participants into risk categories for developing CVDs, we observed an equal pattern between our two groups of participants, with 5.1% having no CVD risk, 12.9% having a mild CVD risk, 15.2% having a moderate CV risk, and 66.8% having a severe CVD risk (Table 1). Due to this and considering the well-known risk factors for kidney diseases in general, age and eGFR, we next examined the correlation pattern between AGE, age, and eGFR when stratifying the participants based on their sex. No difference was found between the sexes; however, we observed a strong positive correlation between the AGE and age, meaning that the older participants have higher levels of AGE, and a strong negative correlation between the AGE and eGFR, meaning that the participants with lower kidney function have higher levels of AGE (Figure 1).

When comparing the nutritional status of the two groups of participants, we did not observe any parameter to be significantly different, except for phase angle, which was higher in the CKD group (median ± IQR, 5.4 ± 1.3 vs. 5.0 ± 1.0, *p* = 0.002), and trunk visceral fat, which was higher in the KTRs (median ± IQR, 10.2 ± 6.1 vs. 6.8 ± 3.7, *p* = 0.002) (Table 2).

When examining the blood pressure parameters, the only significant difference was observed in heart rate, where the CKD group had significantly higher heart rate values compared to the KTRs (median ± IQR, 75 ± 18 vs. 72 ± 17, *p* = 0.037). PWV, a measure of arterial stiffness, was also slightly higher in the CKD group; however, the finding was not statistically significant with this sample size (median ± IQR, 64.0 ± 9.8 vs. 61.1 ± 10.5, *p* = 0.052) (Table 3). Finally, statistically significant differences in medication use were found between the two group of patients. Significantly more KTRs use beta blockers (72.7% vs. 43.0%, *p* < 0.001), alpha1 antagonists (10.1% vs. 0.0%, *p* = 0.002), antihypertensives (87.9% vs. 72.9%, *p* = 0.010), corticosteroids (88.9% vs. 20%, *p* < 0.001), and calcineurin inhibitors (82.8% vs. 2.7%, *p* < 0.001), while more CKD patients use peroral antihyperglycemics (26.3% vs. 11.1%, *p* = 0.008).

**Figure 2:**
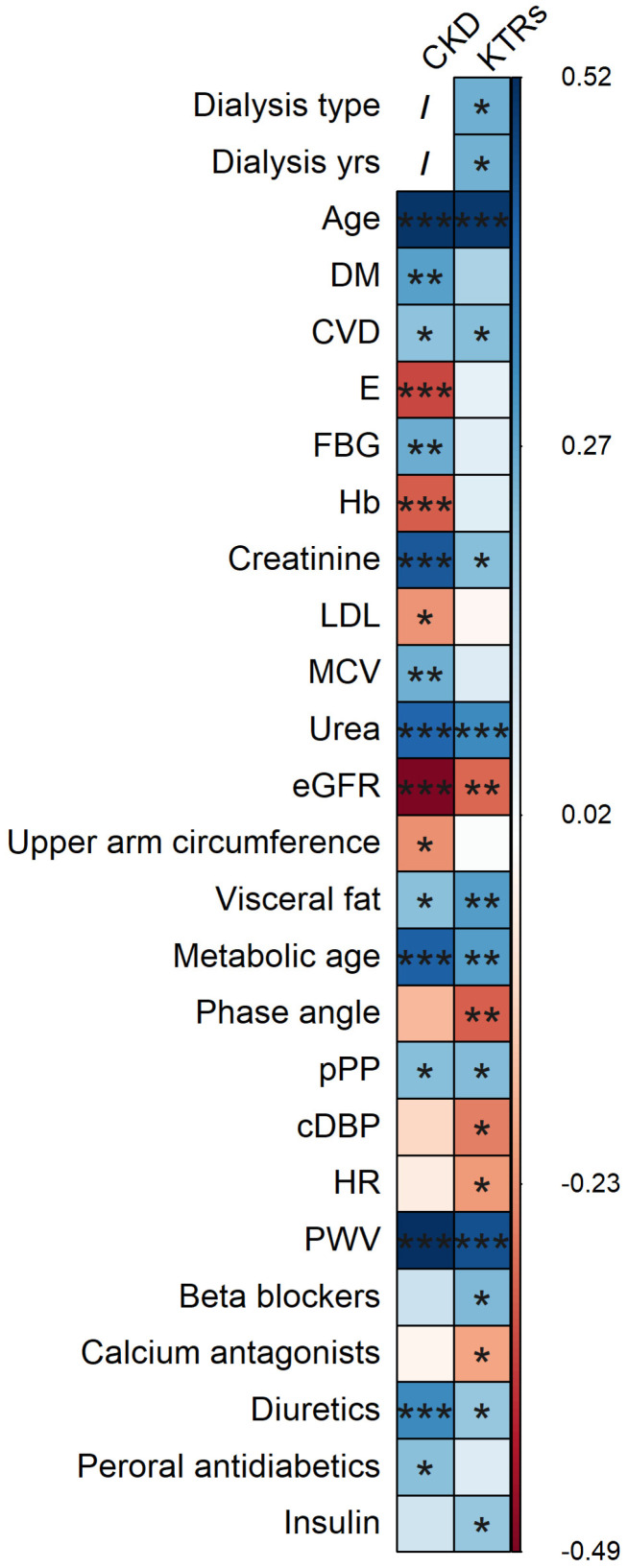
Spearman’s rank correlation plot for statistically significantly correlated parameters and advanced glycation end products (AGE), stratified by kidney disease modality. Abbreviations: CKD—chronic kidney disease, KTRs—kidney transplant recipients, DM—diabetes mellitus, CVD—cardiovascular disease, E—erythrocyte count, FBG—fasting blood glucose (mmol/L), Hb—haemoglobin (g/L), LDL—low-density lipoprotein cholesterol (mmol/L), MCV—mean cellular volume (fL), eGFR—estimated glomerular filtration rate using CKD-EPI (mL/min/1.73 m^2^), pPP—peripheral pulse pressure (mmHg), cDBP—central diastolic blood pressure (mmHg), HR—heart rate (beat/minute), and PWV—pulse wave velocity (m/s). *p*-values labels: *** *p* < 0.001, ** *p* < 0.01, * *p* < 0.05, and none—that correlation was not statistically significant (*p* > 0.05). Cells depicted in red represents negative correlation, while cells depicted in blue represents positive correlation; the deeper the shade the stronger is the correlation coefficient.

To examine the correlation pattern between all the measured variables and AGE, separately for each group of participants, Spearman’s rank correlation analysis was performed, and the summary of the statistically significant results is depicted in Figure 2. In both groups, age, the presence of CVD, creatinine levels, urea levels, levels of visceral fat, metabolic age, pPP, PWV, and use of diuretics were positively correlated with the AGE. Only the eGFR was negatively correlated with the AGE in both groups. In addition, the CKD group showed a significant positive correlation with the AGE and the presence of diabetes mellitus, levels of GUP, levels of MCV, and use of peroral antidiabetics while demonstrating a significant negative correlation between the AGE and levels of E, levels of Hb, levels of LDL, and upper arm circumference. The KTRs group showed an additionally significant positive correlation with the AGE and type of dialysis prior to KTx, years spent on dialysis prior to KTx, use of beta blockers, and insulin while demonstrating a negative correlation with the AGE and levels of phase angle, cDBP, HR, and use of calcium antagonists.

**Figure 3:**
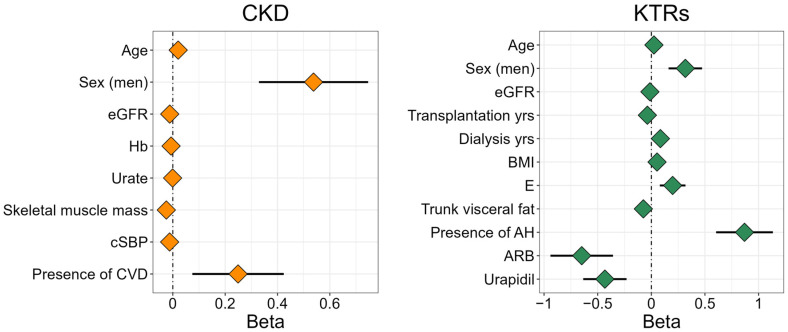
Stepwise linear regression describing the predictors for increased AGE levels. Abbreviations: CKD—chronic kidney disease, KTRs—kidney transplant recipients, eGFR—estimated glomerular filtration rate using CKD-EPI (mL/min/1.73 m^2^), Hb—haemoglobin (g/L), cSBP—central systolic blood pressure (mmHg), CVD—cardiovascular disease, transplantation yrs.—time since kidney transplantation (years), dialysis yrs.—dialysis duration prior to kidney transplantation (years), BMI—body mass index (kg/m^2^), E—erythrocyte count, AH—arterial hypertension, and ARB—angiotensin receptor blocker.

Due to the many measured parameters and the high correlations among them, we first performed a univariate linear regression adjusted for age, sex, and eGFR to analyse the influence of different parameters on the AGE levels separately for each group of participants (Supplementary Table S1). To perform the multiple linear regression, we then selected all the parameters with *p*-values < 0.2, excluded those that showed high collinearity between them (variance inflation factor, VIF > 4), and performed a stepwise linear regression using both backward and forward selection. The parameters that remained in the final model represent the predictors for increased AGE levels among the two groups of participants (Table 4, Figure 3). For both groups, increased AGE levels were predicted the most with older age, being male, and having lower eGFR levels. In addition, for the CKD group, increased AGE levels were associated with lower cSBP, while, for the KTRs group, increased AGE levels were influenced by more years spent on dialysis prior to KTx, less time since KTx, lower levels of trunk visceral fat, the presence of arterial hypertension, and if the participants did not use antihypertensive drugs urapidil and angiotensin II receptor blockers.

Finally, the substratification of the patients within the CKD and KTRs groups as per the CKD stage and results on their median AGE values is presented in Supplementary Table S2.

## 4. Discussion

To the best of our knowledge, this is the first study to compare the AGE levels of patients with CKD and KTRs and investigate the risk variables associated with the AGE levels in each patient group. The study’s findings did not demonstrate a statistically significant difference in the two patient groups’ AGE levels, ages, or sexes.

As predicted, the KTRs showed a significant improvement in the laboratory markers showing renal function, such as urea, creatinine, and eGFR, when compared to the patients with CKD [15]. Nevertheless, the levels of AGE molecules remained unusually and disproportionately high relative to the eGFR, suggesting that additional factors, such as increased oxidative stress, may influence the AGE production in the context of KTRs [15]. This, combined with the lack of difference in the AGE levels between the two groups in our study population, indicates that other factors regardless of the eGFR affect the AGE level. It is a well-known fact that increased AGE levels are a result of both increased production due to chronic inflammation, uraemia, and oxidative stress as well as decreased kidney clearance [7]. End-stage renal disease by itself is a condition in which AGE accumulation is a result of nonenzymatic glycation, oxidative stress, and diminished clearance of AGE precursors [24]. One of the main contributors to oxidative stress in these patients is the bio-incompatibility of the dialysis membrane or reduced antioxidant activity [25]. Due to that, it has been suggested that dialysis by itself contributes strongly to increased AGE levels [26]. On that account, patients with CKD, particularly those on renal replacement therapy, are exposed to a considerable degree of metabolic stress.

The presence of arterial hypertension and CVA was significantly higher in KTRs, as expected, considering the cardiovascular risk factors such as hyperlipidaemia, hypertension, and diabetes mellitus as well as CVA diagnoses, which are common in KTRs [2,3]. Other than the traditional risk factors for arterial hypertension, KTRs have additional calcineurin inhibitors use and possible knocking/stenosis of the renal arteries [27]. The higher incidence of arterial hypertension in KTRs together with the elevated calcification of blood vessels as a consequence of dialysis treatment prior to KTx could be an explanation for the higher incidence of CVA in KTRs [28]. A significant difference was not found in the parameters of the central or peripheral blood pressure between CKD and KTRs. The differences in the laboratory findings correlated with the current studies regarding the improvements after KTx [7].

When it comes to antihypertensive therapy, it is important to mention that only 18% of the KTRs population was prescribed angiotensin-converting enzyme inhibitors, which is a first-choice therapy for treating hypertension. This could be due to clinical inertia. The remaining significant differences in terms of pharmacological therapy correlated with the differences in the clinical and pharmacological approaches to these patients, as was expected [29].

Regarding the body composition, the phase angle, a marker of a better nutritional status, was higher in CKD when compared to the KTRs, which could be due to many reasons. As the phase angle reflects the muscle mass as well, the explanation for this difference could be corticosteroid-induced muscle deterioration after KTx [30]. More reasons for this difference could be the quality of the dietary intake in KTRs, an exceptionally low adherence to the Mediterranean diet that was found in previous research, and low levels of physical activity in KTRs [31].

Interestingly, the KTRs had significantly higher trunk fat mass compared to the CKD patients. One of the well-known side effects of KTx is weight gain in the first months following transplantation, which is related to an increase in fat mass, whereas the fat-free mass remains mainly stable [8]. An increase in body weight after KTx may reflect the normalisation of a pre-existing malnourished state but also the removal of dietary restrictions, an improved appetite, and a decrease in physical activity due to transplantation, but, unfortunately, we did not assess physical activity in this study, which could be considered as a limitation of this study [8,9]. Although moderate weight gain during the first year after KTx has been associated with the best outcome, body composition changes have been related to higher rates of hypertension, diabetes, or dyslipidaemia, elevating the risk of graft loss and death in obese KTRs [10]. The body composition parameters may be similar since the time after KTx was more than one year or because both groups of patients tend not to adhere to dietary recommendations, as we found in our previous studies [31,32].

Regarding the predictors for AGE levels, the results showed that, for both groups, increased AGE levels were predicted the most with older age, being male, and having lower eGFR levels. These findings correlate with the current knowledge on the factors for AGE increases. As proposed in Maillard’s theory of ageing by Monnier and Cerami, the slow accumulation of AGE molecules over a long period of time was a causal factor in ageing. More precisely, the AGE build-up could affect the structure and function of proteins and, therefore, influence several ageing features [5]. Up-to-date evidence suggests that AGE are linked to the onset of various age-related morbidities, indicating that these molecules may not just be a biomarker but also a potential driver of ageing [5,6].

When observing the KTRs group, the negative predictors for AGE levels were the time since KTx, levels of trunk visceral fat, and use of antihypertensive drugs urapidil and angiotensin II receptor blockers, while the positive predictors were the years spent on dialysis prior to KTx, presence of arterial hypertension, BMI, and erythrocyte count. Interestingly, the time spent on dialysis prior to KTx, as previously stated, is proven to have a considerable effect on AGE levels [15]. The time since KTx is associated with lower levels of AGE. Furthermore, studies evaluating the AGE levels within the first 6 months after KTx showed that the blood AGE levels decreased by 70–80% [17]. On the contrary, some studies reported disproportionally high blood AGE levels after KTx when related to recovered renal function [18,33]. Therefore, other factors unrelated to renal function may influence the AGE formation after KTx as well. The use of calcineurin inhibitors, especially cyclosporine, has been associated with enhanced oxidative stress and thus might influence the increased AGE levels found in KTRs [34].

Considering pharmacological therapy, in our results, the angiotensin II receptor blockers and urapidil were found to be associated with reduced levels of AGE in KTRs. Drugs such as angiotensin-converting enzyme inhibitors, vitamins, aminoguanidine, statins, and metformin are known to inhibit AGE formation [35]. However, no studies were conducted to examine the influence of urapidil on AGE levels. Some studies showed that urapidil significantly reduces the fasting blood glucose and glycated haemoglobin (HbA1c) during treatment with 60 mg and 120 mg/day dosages. Also, the ratio of insulin to glucose proved to be significantly lower after the treatment with urapidil. All the above aspects combined suggest that urapidil increases insulin sensitivity since these patients needed less endogenous insulin to maintain their blood glucose levels [36]. This positive metabolic effect that urapidil has on glucose levels could be the reason behind the overall findings on the AGE levels in our study.

In observing the CKD group, the negative predictors of AGE were haemoglobin, uric acid, and skeletal muscle mass, while the positive predictor was the presence of CVD. In addition, for the CKD group, increased AGE levels were associated with lower cSBP. Sourris et al. reported that the plasma levels of AGE were inversely related to the diastolic pressure after the adjustment for the age, sex, BMI, and waist–hip ratio in the general population. The AGE levels were positively correlated with pPP. However, there was no correlation between the AGE and pSBP [37]. These differences might be due to differences in the study population. These results may suggest that AGE levels are predictive of an early diagnosis of sarcopenia, which is known to be a severe burden in the CKD population. This association needs to be better understood and requires further prospective studies.

This study has some limitations that need to be addressed. The main limitation arises from the cross-sectional design of the study, which can determine only association but not causality. Physical activity and drug dosage were also not considered when conducting this study. Another limitation of this study is that we did not take into consideration the etiological background of CKD due to the availability of data and late informatisation of the healthcare system.

Evaluating AGE levels is potentially a valuable asset in CKD and KTRs evaluation and treatment courses. However, further studies are needed to better understand the above associations, especially in terms of a randomised design to also assess the causality. The results of this study contribute to the understanding of AGE values and their potential in clinical nephrology. In conclusion, a personalised multidisciplinary approach is undoubtedly needed to assess the cardiovascular as well as dietary and lifestyle risk factors to reduce the AGE levels in both KTRs and CKD patients.

## Figures and Tables

**Table 1:** General characteristics of study participants and differences between CKD and KTRs groups of participants.

		Total (*N* = 217)	CKD (*N* = 118)	KTRs (*N* = 99)	*p* *
Age (years), median (IQR)		64 (18)	64 (20.25)	64 (16.5)	0.665
Sex, *N* (%)	Women	86 (39.63)	47 (39.83)	39 (39.39)	1.000
	Men	131 (60.37)	71 (60.17)	60 (60.61)	
AGE, median (IQR)		3.3 (1.3)	3.3 (1.5)	3.2 (1.25)	0.548
AGE categories, *N* (%)	No CVD risk	11 (5.07)	5 (4.24)	6 (6.06)	0.897
	Mild CVD risk	28 (12.9)	15 (12.71)	13 (13.13)	
	Moderate CVD risk	33 (15.21)	17 (14.41)	16 (16.16)	
	Severe CVD risk	145 (66.82)	81 (68.64)	64 (64.65)	
COMORBIDITIES					
Presence of arterial hypertension, *N* (%)	No	43 (19.91)	33 (28.21)	10 (10.1)	0.002
	Yes	173 (80.09)	84 (71.79)	89 (89.9)	
Presence of diabetes mellitus, *N* (%)	No	154 (70.97)	78 (66.1)	76 (76.77)	0.115
	Yes	63 (29.03)	40 (33.9)	23 (23.23)	
Presence of CVD, *N* (%)	No	156 (72.22)	86 (73.5)	70 (70.71)	0.760
	Yes	60 (27.78)	31 (26.5)	29 (29.29)	
Presence of CVA, *N* (%)	No	202 (93.09)	115 (97.46)	87 (87.88)	0.012
	Yes	15 (6.91)	3 (2.54)	12 (12.12)	
LABORATORY PARAMETERS					
Urea (mmol/L), median (IQR)		11.95 (12.9)	18 (16.3)	9.8 (5.85)	<0.001
Creatinine (mmol/L), median (IQR)		159 (183)	260 (322)	132 (57.25)	<0.001
eGFR (mL/min/1.73 m^2^), median (IQR)		36.15 (37.73)	17.65 (31.65)	43.45 (29.1)	<0.001
E (×10^12^/L), mean (SD)		4.33 (0.77)	4.04 (0.71)	4.67 (0.7)	<0.001
Hb (g/L), mean (SD)		126.22 (19.33)	121.41 (19.57)	131.78 (17.58)	<0.001
MCV (fL), median (IQR)		88.1 (7.45)	88.95 (7.95)	87.2 (7)	0.017
CRP (mg/L), median (IQR)		2.9 (5.4)	3.75 (7.2)	2.2 (3.95)	0.040
Na (mmol/L), median (IQR)		140 (4)	140 (4)	141 (3)	0.001
K (mmol/L), median (IQR)		4.3 (0.7)	4.5 (0.7)	4.1 (0.6)	<0.001
Ca (mmol/L), median (IQR)		2.34 (0.19)	2.3 (0.18)	2.41 (0.16)	<0.001
P (mmol/L), median (IQR)		1.12 (0.41)	1.25 (0.49)	1.02 (0.25)	<0.001
FBG (mmol/L), median (IQR)		5.4 (1.45)	5.5 (2)	5.3 (1.05)	0.058
Total cholesterol (mmol/L), median (IQR)		5 (1.6)	4.6 (1.9)	5.5 (1.5)	0.001
LDL (mmol/L), median (IQR)		2.8 (1.4)	2.5 (1.6)	3.1 (1.36)	0.002
Tgl (mmol/L), median (IQR)		1.6 (1.19)	1.5 (1.1)	1.75 (1.22)	0.370
Uric acid (mmol/L), median (IQR)		391 (120)	392 (128)	385.5 (107.5)	0.667
Alb (g/L), median (IQR)		41.1 (5.6)	40.2 (5.5)	41.7 (4.38)	0.013

* *p*-values were obtained with chi-squared test for categorical data, *t*-test for parametric numerical data, and Mann–Whitney U test for non-parametric numerical data. Abbreviations: CKD—chronic kidney disease, KTRs—kidney transplant recipients, AGE—advanced glycation end products, CVD—cardiovascular disease, CVA—cerebrovascular disease, eGFR—estimated glomerular filtration rate using CKD-EPI (mL/min/1.73 m^2^), E—erythrocyte count, Hb—haemoglobin (g/L), MCV—mean cellular volume (fL), CRP—C-reactive protein (mg/L), Na—sodium (mmol/L), K—potassium (mmol/l), Ca—calcium (mmol/L), P—phosphates (mmol/L), FBG—fasting blood glucose (mmol/L), LDL—low-density lipoprotein cholesterol (mmol/L), Tgl—triglycerides (mmol/L), and Alb—serum albumin (g/L).

**Table 2:** Nutritional status of study participants and differences between CKD and KTRs groups of participants.

		Total (*N* = 217)	CKD (*N* = 118)	KTRs (*N* = 99)	*p* *
ANTHROPOMETRIC PARAMETERS					
Height (cm), mean (SD)		173.8 (10.08)	173.47 (10.07)	174.22 (10.14)	0.589
Weight (kg), median (IQR)		78.6 (22.7)	77.2 (26.2)	79.8 (20.58)	0.394
BMI (kg/m^2^), mean (SD)		25.7 (5.85)	25.25 (6.17)	26.15 (5.33)	0.220
Middle upper arm circumference (cm), median (IQR)		30 (6)	29 (5.5)	31 (5)	0.055
Waist circumference (cm), mean (SD)		97.68 (13.01)	96.66 (13.85)	99.02 (11.75)	0.209
WHtR, mean (SD)		0.56 (0.09)	0.55 (0.08)	0.56 (0.08)	0.216
BODY COMPOSITION					
Fat mass (kg), median (IQR)		17.4 (12)	15.65 (11.7)	19.4 (11.1)	0.058
Fat mass (%), mean (SD)		22.8 (9.08)	21.91 (9.14)	23.86 (8.94)	0.128
Fat-free mass (kg), median (IQR)		60.6 (18.85)	61.4 (19.92)	60 (17.6)	0.746
Visceral fat, median (IQR)		9 (5)	8 (6)	9 (5)	0.216
Metabolic age (years), median (IQR)		52 (16)	52 (17.5)	52 (15)	0.910
Muscle mass (kg), median (IQR)		57.6 (17.95)	58.35 (19.02)	57 (16.8)	0.745
Skeletal muscle mass (kg), median (IQR)		32.2 (11.4)	32.9 (11.38)	32 (11.4)	0.570
Skeletal muscle mass (%), median (IQR)		42.5 (9.35)	43.05 (9.45)	41.1 (10)	0.079
Phase angle (◦), median (IQR)		5.2 (1.2)	5.4 (1.27)	5 (1)	0.002
Trunk visceral fat, median (IQR)		9.7 (6.45)	6.8 (3.7)	10.2 (6.1)	0.004

* *p*-values were obtained with chi-squared test for categorical data, *t*-test for parametric numerical data, and Mann–Whitney U test for non-parametric numerical data. Abbreviations: CKD—chronic kidney disease, KTRs—kidney transplant recipients, BMI—body mass index, and WHtR—weight-to-height ratio.

**Table 3:** Blood pressure levels and use of medications of study participants and differences between CKD and KTRs groups of participants.

		Total (*N* = 217)	CKD (*N* = 118)	KTRs (*N* = 99)	*p* *
BLOOD PRESSURE PARAMETERS					
pSBP (mmHg), median (IQR)		137 (29.5)	141.5 (32)	135 (20.5)	0.069
pDBP (mmHg), mean (SD)		88.29 (12.6)	88.3 (12.77)	88.27 (12.47)	0.989
pMAP (mmHg), median (IQR)		112 (20.25)	112.5 (21.75)	112 (18.75)	0.545
pPP (mmHg), median (IQR)		53 (22)	55 (19)	50 (22)	0.120
cSBP (mmHg), median (IQR)		126.25 (26.25)	126 (27)	126.5 (21)	0.759
cDBP (mmHg), mean (SD)		89.74 (13.05)	89.87 (12.94)	89.55 (13.27)	0.876
cMAP (mmHg), median (IQR)		101.58 (15.25)	100 (13.83)	102 (14.67)	0.478
cPP (mmHg), median (IQR)		37 (16.12)	38 (14.5)	36.5 (15)	0.270
HR (beat/min), median (IQR)		74 (17)	75 (18)	72 (17)	0.037
Aix (%), median (IQR)		23 (22.75)	24 (24)	20.5 (22)	0.131
PWV (m/s), median (IQR)		62.67 (10.18)	63.99 (9.75)	61.09 (10.51)	0.052
MEDICATION USE					
Beta blockers, *N* (%)	No	92 (43.19)	65 (57.02)	27 (27.27)	<0.001
	Yes	121 (56.81)	49 (42.98)	72 (72.73)	
Angiotensin-converting enzyme inhibitors, *N* (%)	No	160 (75.12)	79 (69.3)	81 (81.82)	0.051
	Yes	53 (24.88)	35 (30.7)	18 (18.18)	
Angiotensin II receptor blockers, *N* (%)	No	200 (93.9)	108 (94.74)	92 (92.93)	0.793
	Yes	13 (6.1)	6 (5.26)	7 (7.07)	
Calcium channel blockers, *N* (%)	No	87 (40.85)	50 (43.86)	37 (37.37)	0.412
	Yes	126 (59.15)	64 (56.14)	62 (62.63)	
Alpha1 antagonists, *N* (%)	No	203 (95.31)	114 (100)	89 (89.9)	0.002
	Yes	10 (4.69)	NA	10 (10.1)	
Aldosterone antagonist, *N* (%)	No	210 (98.59)	113 (99.12)	97 (97.98)	0.902
	Yes	3 (1.41)	1 (0.88)	2 (2.02)	
Moxonidine, *N* (%)	No	132 (61.97)	69 (60.53)	63 (63.64)	0.745
	Yes	81 (38.03)	45 (39.47)	36 (36.36)	
Diuretics, *N* (%)	No	72 (33.8)	39 (34.21)	33 (33.33)	1.000
	Yes	141 (66.2)	75 (65.79)	66 (66.67)	
Peroral antihyperglycemics, *N* (%)	No	172 (80.75)	84 (73.68)	88 (88.89)	0.008
	Yes	41 (19.25)	30 (26.32)	11 (11.11)	
Insulin, *N* (%)	No	179 (84.43)	98 (86.73)	81 (81.82)	0.333
	Yes	32 (15.09)	14 (12.39)	18 (18.18)	
Statins, *N* (%)	No	126 (59.43)	73 (64.6)	53 (53.54)	0.134
	Yes	86 (40.57)	40 (35.4)	46 (46.46)	
Urapidil, *N* (%)	No	183 (85.92)	101 (88.6)	82 (82.83)	0.313
	Yes	30 (14.08)	13 (11.4)	17 (17.17)	
Antihypertensives, *N* (%)	No	44 (20.28)	32 (27.12)	12 (12.12)	0.010
	Yes	173 (79.72)	86 (72.88)	87 (87.88)	
Corticosteroids, *N* (%)	No	103 (48.13)	92 (80)	11 (11.11)	<0.001
	Yes	111 (51.87)	23 (20)	88 (88.89)	
Calcineurin inhibitors, *N* (%)	No	127 (59.91)	110 (97.35)	17 (17.17)	<0.001
	Yes	85 (40.09)	3 (2.65)	82 (82.83)	

* *p*-values were obtained with the chi-squared test for categorical data, *t*-test for parametric numerical data, and Mann–Whitney U test for non-parametric numerical data. Abbreviations: CKD—chronic kidney disease, KTRs—kidney transplant recipients, pSBP—peripheral systolic blood pressure, pDBP—diastolic blood pressure, pMAP—peripheral mean arterial pressure, pPP—peripheral pulse pressure, cSBP—central systolic blood pressure, cDBP—central blood pressure, cMAP—central mean arterial pressure, cPP—central pulse pressure, HR—heart rate, Aix—augmentation index, and PWV—pulse wave velocity.

**Table 4:** Results of stepwise linear regression describing the predictors for increased AGE levels.

Predictor	Beta	SE	*p*
Predictors for CKD (R^2^ = 44.1%)			
Age (years)	0.020	0.006	<0.001
Sex (men)	0.538	0.209	0.011
eGFR (mL/min/1.73 m^2^)	−0.012	0.003	<0.001
Hb (g/L)	−0.007	0.004	0.126
Uric acid (mmol/L)	−0.001	0.001	0.170
Skeletal muscle mass (kg)	−0.025	0.013	0.055
cSBP (mmHg)	−0.012	0.004	0.005
Presence of CVD	0.249	0.175	0.156
**Predictors for KTRs (R^2^ = 46.9%)**			
Age (years)	0.024	0.006	<0.001
Sex (men)	0.317	0.156	0.045
eGFR (mL/min/1.73 m^2^)	−0.013	0.004	0.002
Time since KTx (years)	−0.037	0.014	0.010
Dialysis duration prior to KTx (years)	0.085	0.020	<0.001
BMI (kg/m^2^)	0.053	0.033	0.115
E (×10^12^/L)	0.199	0.120	0.100
Trunk fat mass (kg)	−0.074	0.027	0.007
Presence of AH	0.869	0.265	0.001
Angiotensin II receptor blockers	−0.649	0.292	0.029
Urapidil	−0.433	0.202	0.035

Abbreviations: AGE—advanced glycation end products, CKD—chronic kidney disease, KTRs—kidney transplant recipients, eGFR—estimated glomerular filtration rate using CKD-EPI (mL/min/1.73 m^2^), Hb—haemoglobin (g/L), cSBP—central systolic blood pressure, CVD—cardiovascular disease, KTx—kidney transplantation, BMI—body mass index (kg/m^2^), E—erythrocyte count, and AH—arterial hypertension.

## Data Availability

Data are available upon request at the corresponding author e-mail.

## Supplementary Materials

The following supporting information can be downloaded at https://www.mdpi.com/article/10.3390/diagnostics14131383/s1, Table S1: Influence of different parameters on AGE levels, separately for each group of participants. Table S2: Substratification of patients within CKD and KTRs groups as per CKD stage and results on their median AGE values.
