# Supplementary material for: Are There Differences in Skin Autofluorescence-Measured Advanced Glycation End-Product Levels between Chronic Kidney Disease and Kidney Transplant Recipients?
Source: Diagnostics (Basel). 2024 Jun 28;14(13):1383. doi: 10.3390/diagnostics14131383 (PMC11240785; doi:10.3390/diagnostics14131383)
Supplement: Supplementary file 1 [file diagnostics-14-01383-s001.zip › diagnostics-3050883-supplementary.pdf]

Supplementary Table S1. Results of univariate linear regression adjusted for age, gender and eGFR describing the contribution of each measured parameter to AGE levels

	**CKD (N = 118)**			**KTRs (N = 99)**		
	Beta	SE	p	Beta	SE	p
Time since KTx (years)	/			-0.022	0.015	0.143
Dialysis duration prior KTx (years)	/			0.067	0.022	0.003
COMORBIDITIES						
Presence of arterial hypertension	-0.004	0.198	0.984	0.445	0.301	0.142
Presence of diabetes mellitus	0.102	0.188	0.587	0.023	0.206	0.910
Presence of CVD	0.260	0.190	0.174	0.182	0.197	0.359
Presence of CVA	-0.147	0.525	0.781	0.167	0.258	0.521
LABORATORY PARAMETERS						
Alb (g/L)	0.009	0.016	0.559	0.013	0.031	0.674
Ca (mmol/L)	0.026	0.516	0.960	0.231	0.432	0.595
CRP (mg/L)	0.000	0.003	0.933	0.011	0.015	0.472
E (x10 12 /L),	-0.182	0.138	0.192	0.204	0.137	0.139
FBG (mmol/L)	-0.005	0.047	0.922	-0.053	0.076	0.488
Hb (g/L)	-0.008	0.005	0.109	0.008	0.006	0.179
K (mmol/L)	-0.113	0.138	0.416	-0.111	0.191	0.563
Total cholesterol (mmol/L)	-0.096	0.064	0.133	-0.132	0.090	0.144
Creatinine (mmol/L)	0.000	0.000	0.549	-0.002	0.003	0.481
LDL (mmol/L)	-0.153	0.083	0.068	-0.082	0.096	0.399
MCV (fL)	0.013	0.010	0.189	-0.001	0.015	0.973
Na (mmol/L)	-0.036	0.029	0.220	0.023	0.035	0.509
P (mmol/L)	0.159	0.271	0.558	-0.786	0.444	0.080
Tgl (mmol/L)	0.010	0.075	0.898	-0.084	0.091	0.356
Uric acid (mmol/L)	-0.001	0.001	0.090	0.001	0.001	0.240
Urea (mmol/L)	0.008	0.013	0.528	0.002	0.025	0.927
ANTHROPOMETRIC PARAMETERS						
Height (cm)	-0.014	0.013	0.279	-0.021	0.012	0.067
Weight (kg)	-0.012	0.005	0.011	-0.009	0.006	0.161
BMI (kg/m^2), mean (SD)	-0.036	0.016	0.024	-0.016	0.021	0.452
Middle upper arm circumference (cm)	-0.022	0.010	0.025	-0.004	0.008	0.580
Waist circumference (cm)	-0.010	0.007	0.142	-0.006	0.007	0.371
WHtR	-1.466	1.149	0.205	-0.361	1.181	0.760
BODY COMPOSITION						
Fat mass (kg)	-0.015	0.008	0.070	-0.003	0.010	0.730
Fat mass (%)	-0.014	0.011	0.206	-0.001	0.011	0.920
Fat-free mass (kg)	-0.021	0.009	0.019	-0.010	0.011	0.334
Visceral fat	-0.034	0.027	0.214	-0.011	0.028	0.694
Metabolic age	0.000	0.010	0.966	-0.008	0.010	0.421
Muscle mass (kg)	-0.022	0.009	0.018	-0.011	0.011	0.335
Skeletal muscle mass (kg)	-0.030	0.014	0.034	-0.009	0.017	0.577
Skeletal muscle mass (%)	0.026	0.015	0.091	0.001	0.015	0.973
Phase angle (◦)	0.045	0.066	0.493	0.010	0.141	0.943

	CKD (N = 118)			KTRs (N = 99)		
	Beta	SE	p	Beta	SE	p
Trunk visceral fat	-0.024	0.047	0.620	-0.026	0.016	0.108
BLOOD PRESSURE PARAMETERS						
pSBP (mmHg)	-0.010	0.004	0.025	-0.001	0.004	0.805
pDBP (mmHg)	-0.021	0.007	0.002	-0.002	0.007	0.738
pMAP (mmHg)	-0.015	0.006	0.008	-0.003	0.006	0.649
pPP (mmHg)	-0.004	0.007	0.582	0.000	0.006	0.949
cSBP (mmHg)	-0.013	0.005	0.012	-0.006	0.005	0.250
cDBP (mmHg)	-0.020	0.007	0.005	-0.009	0.007	0.250
cMAP (mmHg)	-0.006	0.012	0.624	-0.008	0.007	0.221
cPP (mmHg)	-0.008	0.009	0.358	-0.006	0.009	0.475
HR (beat/min)	0.005	0.007	0.471	0.006	0.009	0.495
Aix (%)	0.003	0.007	0.672	-0.004	0.007	0.577
PWV (m/s)	0.018	0.094	0.848	-0.052	0.127	0.681
MEDICATION USE						
Beta blockers	0.121	0.173	0.488	0.169	0.203	0.406
ACEI	-0.011	0.192	0.953	-0.376	0.223	0.096
Angiotensin II receptor blockers	-0.152	0.377	0.688	-0.747	0.324	0.023
Calcium antagonists	-0.380	0.169	0.027	-0.236	0.179	0.190
Alpha1 antagonists	NA	NA	NA	-0.330	0.280	0.242
Aldosteron antagonists	1.581	0.891	0.079	-0.203	0.604	0.737
Moxonidin	-0.404	0.177	0.025	-0.318	0.174	0.071
Diuretics	0.062	0.204	0.761	0.197	0.188	0.297
Peroral antihyperglycemics	-0.054	0.202	0.790	0.042	0.273	0.878
Insulin	-0.093	0.260	0.722	0.137	0.225	0.543
Statins	-0.004	0.183	0.984	-0.009	0.183	0.960
Urapidil	0.046	0.275	0.869	-0.458	0.228	0.047
Antihypertensives	0.016	0.199	0.935	0.371	0.275	0.181
Corticosteroids	0.239	0.216	0.271	0.116	0.282	0.682
Calcineurin inhibitors	0.214	0.551	0.698	0.038	0.231	0.871

Abbreviations: KTx – kidney transplantation, CKD – chronic kidney disease, KTRs – kidney transplant recipients, AGE – advanced glycation end products, CVD – cardiovascular disease, CVA – cerebrovascular disease, eGFR – estimated glomerular filtration rate using CKD-EPI (ml/min/1.73m2), Alb – serum albumin (g/L), Ca – calcium (mmol/L), CRP – C-reactive protein (mg/L), E – erythrocyte count, FBG – fasting blood glucose (mmol/L), Hb – haemoglobin (g/L), K – potassium (mmol/l), LDL – low-density lipoprotein cholesterol (mmol/L), MCV – mean cellular volume (fL), Na – sodium (mmol/L), P – phosphates (mmol/L), Tgl – triglycerides (mmol/L), BMI – body mass index, WHtR – weight-to-height ratio, pSBP – peripheral systolic blood pressure, pDBP – dyastolic blood pressure, pMAP – peripheral mean arterial pressure, pPP – peripheral pulse pressure, cSBP – central systolic blood pressure, cDBP – central blood pressure, cMAP – central mean arterial pressure, cPP – central pulse pressure, HR – heart rate, Aix – augumentation index, PWV – pulse wave velocity, ACEI - Angiotensin - converting enzyme inhibitors

Supplementary Table S2. Substratification of patients within CKD and KTR groups as per CKD stage and results on their median AGE values

CKD stage	CKD (N, %)	KTRs (N, %)	p	CKD AGE value (median, IQR)	KTRs AGE value (median, IQR)	p
**Stage 1**	10 (8.77%)	4 (4.08%)		1.8 (0.35)	2.8 (0.525)	
**Stage 2**	8 (7.02%)	26 (26.53%)		2.55 (1.35)	2.75 (0.7)	
**Stage 3a**	8 (7.02%)	16 (16.33%)	<0.001	3.05 (0.625)	3.05 (0.925)	0.578
**Stage 3b**	16 (14.04%)	37 (37.76%)		3.25 (1.05)	3.6 (1.2)	
**Stage 4**	20 (17.54%)	13 (13.27%)		3.25 (1.125)	3.5 (1.2)	
**Stage 5**	52 (45.61%)	2 (2.04%)		3.5 (1.65)	3.45 (0.45)	

Abbreviations: CKD – chronic kidney disease, KTRs – kidney transplant recipients, AGE – advanced glycation end products
